# Single Cell Genomics-Based Analysis of Gene Content and Expression of Prophages in a Diffuse-Flow Deep-Sea Hydrothermal System

**DOI:** 10.3389/fmicb.2019.01262

**Published:** 2019-06-12

**Authors:** Jessica M. Labonté, Maria Pachiadaki, Elizabeth Fergusson, Jesse McNichol, Ashley Grosche, Lara K. Gulmann, Costantino Vetriani, Stefan M. Sievert, Ramunas Stepanauskas

**Affiliations:** ^1^Bigelow Laboratory for Ocean Sciences, East Boothbay, ME, United States; ^2^Department of Marine Biology, Texas A&M University at Galveston, Galveston, TX, United States; ^3^Biology Department, Woods Hole Oceanographic Institution, Woods Hole, MA, United States; ^4^Department of Biochemistry and Microbiology, Rutgers University, New Brunswick, NJ, United States; ^5^Department of Marine and Coastal Sciences, Rutgers University, New Brunswick, NJ, United States

**Keywords:** prophages, single cell genomics, phage life cycle, hydrothermal vent, lysogeny

## Abstract

Phage–host interactions likely play a major role in the composition and functioning of many microbiomes, yet remain poorly understood. Here, we employed single cell genomics to investigate phage–host interactions in a diffuse-flow, low-temperature hydrothermal vent that may be reflective of a broadly distributed biosphere in the subseafloor. We identified putative prophages in 13 of 126 sequenced single amplified genomes (SAGs), with no evidence for lytic infections, which is in stark contrast to findings in the surface ocean. Most were distantly related to known prophages, while their hosts included bacterial phyla Campylobacterota, Bacteroidetes, Chlorobi, Proteobacteria, Lentisphaerae, Spirochaetes, and Thermotogae. Our results suggest the predominance of lysogeny over lytic interaction in diffuse-flow, deep-sea hydrothermal vents, despite the high activity of the dominant Campylobacteria that would favor lytic infections. We show that some of the identified lysogens have co-evolved with their host over geological time scales and that their genes are transcribed in the environment. Functional annotations of lysogeny-related genes suggest involvement in horizontal gene transfer enabling host’s protection against toxic metals and antibacterial compounds.

## Introduction

Hydrothermal vents represent oases of high biological productivity in the deep-sea and are the first environments where chemolithoautotrophy was identified as a primary source of organic carbon production (Corliss et al., 1979; Jannasch and Mottl, 1985). Deep-sea hydrothermal vents are characterized by the emission of chemically-altered fluids that form through seawater–rock interactions at high temperature and pressure, resulting in fluids that are enriched in reduced chemical species such as H_2_, H_2_S, Fe^2+^, and CH_4_, with the composition and concentration strongly dependent upon the geological setting (Kelley et al., 2002). The mixing of high-temperature hydrothermal fluids with seawater creates gradients of pH, temperature and chemicals, both above and below the seafloor (Baross and Hoffman, 1985), generating microenvironments suitable for diverse microbial communities (Huber et al., 2003; Schrenk et al., 2003). Vent prokaryotes have adapted unique strategies to survive under these dynamic physical–chemical conditions (Nakagawa and Takai, 2008), likely dating back to the Archaean (Kelley et al., 2002). Metagenomic studies of diffuse-flow deep-sea vents have revealed a high proportion of genes related to prophages and horizontal gene transfer (Anderson et al., 2011a,b). Additional evidence for the higher abundance of prophages in hydrothermal systems, as compared to the surrounding deep-sea water, has been provided by the experimental induction of prophages with mitomycin C (Williamson et al., 2008). These studies suggest that viruses in general, and temperate phages in particular, may play important roles in the ecology and evolution of microorganisms in hydrothermal systems. However, due to the technical challenges of cultivation-based isolation of the predominant lineages of microorganisms and their infecting agents, our current understanding of the phage–host interactions in hydrothermal systems remains rudimentary.

Phages can follow diverse life cycles. They can enter the lytic cycle, releasing virions following cell lysis (virulent phage), and some can enter the lysogenic cycle (temperate phage), in which case their genome becomes integrated inside the one of their hosts. Given the right conditions, functional prophages can then re-enter the lytic cycle. Other life strategies such as chronic infections and pseudolysogeny exist but remain poorly understood. Phages, especially temperate phages, can serve as vectors for the horizontal transfer of genes between bacterial species or even genera via transduction (Canchaya et al., 2003). By establishing a quasi-stable relationship with their hosts through lysogeny, temperate phages may benefit the host population by increasing the host fitness through the expression of new phenotypes, a process called lysogenic conversion (Levin and Lenski, 1983). Other advantages of carrying prophages may include the immunity of the host against phage infections of related viruses through the prophage repressor and superinfection exclusion genes (Canchaya et al., 2003). Lysogeny may be very common among isolated strains of bacteria, with an estimated 60–70% of sequenced genomes containing prophage-like sequences (Casjens, 2003; Paul, 2008). In environmental samples, the proportion of lysogens, or bacterial genomes where prophages have been identified in, highly depends on the environment and varies between 0 and 100% (Paul, 2008).

To gain a better understanding of the hosts, genomic content, and roles of phages and prophages in diffuse-flow hydrothermal systems, we analyzed genomic sequences of 126 single amplified genomes (SAGs) of Bacteria and Archaea from the Crab Spa vent on the East Pacific Rise at 9°N (McNichol et al., 2016, 2018). We identified 13 phage sequences and found no evidence for lytic infections, suggesting that they are all prophages. The genome content of the identified prophages suggests complex evolutionary histories and potential roles in the adaptation of their hosts to the dynamic hydrothermal vent environment.

## Materials and Methods

### Generation and Sequencing of Single Amplified Genomes (SAGs)

Fluid samples were collected from Crab Spa (9°50.3981 N, 104°17.4942 W), a diffuse-flow deep-sea hydrothermal vent located on the East Pacific Rise at a water depth of 2,506 m (McNichol et al., 2016, 2018), during oceanographic cruises AT15-38 (2008) and AT26-10 (2014). In 2008, vent fluids for SAG analyses were collected with titanium water samplers (Von Damm et al., 1985). In 2014, additional batch incubations under simulated *in situ* conditions were performed with isobaric gas-tight samplers (Seewald et al., 2001) after amendments with nitrate, hydrogen and/or oxygen at either *in situ* temperature or 50°C (McNichol et al., 2016, 2018). SAGs of planktonic Bacteria and Archaea were generated [using cell sorting and multiple displacement amplification (MDA)] and identified (via sequencing of their SSU rRNA genes) from un-manipulated field samples (2008, 2014) and from isobaric incubations (2014), following previously described procedures (Stepanauskas et al., 2017). A total of 126 SAGs were selected for genomic sequencing as follows (see Supplementary Table 1): (a) 20 *Sulfurovum* SAGs with identical SSU rRNA gene sequences (defined as “*Sulfurovum* population”) belonging the class Campylobacteria (formerly Epsilonproteobacteria, Waite et al., 2017, 2018) – the predominant class of bacteria in this and many other hydrothermal vents (Sievert and Vetriani, 2012; McNichol et al., 2018); (b) 26 SAGs representing a wide spectrum of Campylobacteria (defined as “Diverse Campylobacteria”); (c) 32 SAGs representing other, phylogenetically diverse lineages of bacteria and archaea that are known to be ubiquitous in hydrothermal vents or other subsurface environments (defined as “Other community members”); and (d) 48 SAGs representing deep branches of the tree of life with no or few cultured representatives (defined as “Microbial dark matter”). These SAGs were sequenced using Illumina technology and *de novo* assembled as previously described (Stepanauskas et al., 2017). The generation, identification, and genomic sequencing of SAGs were performed at Bigelow Laboratory Single Cell Genomics Center.^1^

### Phage Identification

Phage contigs were identified using a combination of viral marker genes [viral genes and families (^∗^phage, ^∗^virus, virion, prophage, terminase, capsid, head, tail, fiber, baseplate, portal, lysis, structural, T4, lambda, mu, lambdoid, podo^∗^, myovir^∗^, siphovir^∗^ excluding integrase and transposase as they are also found in bacterial genomes and can be unrelated to phages)] tRNAs (which are common prophage integration sites), DNA sequence anomalies (GC skew and tetramer frequencies), and metagenomic fragment recruitment from viral and bacterial metagenomes, as previously described (Labonté et al., 2015b). The identified viral contigs were then manually confirmed using diverse comparative genomics tools (Labonté et al., 2015b). To obtain additional evidence, when possible, whole genome synteny comparisons were performed with EasyFig for Mac version 2.1 (Sullivan et al., 2011), with tBLASTx and the filtering of small hits and annotations option.

### Phylogenetic Analyses

For the phage portal protein genes, similar sequences were recovered from the GenBank nr database (E-value < 10^-5^; identity > 35%). Sequences were aligned with MUSCLE (Edgar, 2004) implemented in Geneious V6.1.8. Phylogeny was performed using PhyML V3.1 (Guindon et al., 2010) with 100 bootstrap replicates and the LG model with a gamma distribution (+G), estimated rates of variation among sites and a proportion of invariable sites (+I). For the terminase gene, the sequences were recovered from the GenBank nr database, and aligned based on their amino acid charges with PROMALS (Pei et al., 2008). Phylogeny was performed using PhyML implemented in Geneious with 100 approximate likelihood ratio test (aLTR) replicates and the LG model with a gamma distribution (+G), estimated rates of variation among sites and a proportion of invariable sites (+I). For the SSU rRNA phylogeny, SSU rRNA sequences were first compared with previously deposited sequences using the RDP v10 Classifier (SSU rRNA) (Cole et al., 2014). Using SINA (Pruesse et al., 2012), the SAG SSU rRNA gene sequences were aligned with sequences selected with the RDP Seqmatch (SSU rRNA gene sequences from isolates, ≥1,200 bp of good quality) from the RDP pipeline (Cole et al., 2014) using the rRNA SILVA reference alignment. A maximum likelihood tree of the SAG SSU rRNA gene sequences (100 bootstrap replicates) was constructed using phyML v3.1 (Guindon et al., 2010) with the best model [GTR model with a gamma distribution (+G), estimated rates of variation among sites and a proportion of invariable sites (+I)].

### Average Nucleotide Identity Calculations

For the host genomes, average nucleotide identity (ANI) was calculated using JSpecies (Richter and Rosselló-Móra, 2009) with the ANIb parameters. Because the prophage genomes were too short for JSpecies, the genomes were aligned with the Geneious Aligner with a cost matrix of 65% similarity (Kearse et al., 2012) and the ANI was calculated as the percentage of identity in the distance matrix.

### Large Volume Pump (LVP) Sampling

Filtration of hydrothermal vent fluids was carried out *in situ* with McLane large volume pump (LVP) (McLane, Falmouth, MA, United States) equipped with a hose and nozzle to collect biomass for subsequent “omic” analyses from the diffuse-flow vent sites Crab Spa (LVP2 and LVP4, 9°50.3981 N, 104°17.4942 W) and Teddy Bear (LVP5 and LVP8, 9°50.0 N, 104°17.51 W) during the AT26-10 cruise in 2014. The LVP was dropped as a free-falling instrument from R/V *Atlantis*. Once on the seafloor, it was moved by ROV *Jason* and came back to the surface within 40 min where it was picked up and brought on deck. Once on deck, the filters were taken out of the thermally-insulated filter housing, cut in sections, and stored at -80°C (the filter sections for RNA were soaked in RNAlater overnight at 4°C prior to freezing). The time interval between the end of the pumping and preserving the filter on board the ship was at most 2 h. The amount of hydrothermal fluid pumped through the filter was 422, 345, 326, and 257 l for LVP2, 4, 5, and 8, respectively.

### DNA Extraction From Large Volume Pump Filters

DNA was extracted from one ¼ (1/8 for LVP4) of the LVP filters (GF-75 combusted glass fiber filter with a pore size of 0.3 μm on top of polyethersulfone membrane filter with a pore size of 0.2 μm) using a phenol:chloroform:isoamyl alcohol and chloroform extraction method (Green and Sambrook, 2017). Total DNA was extracted from LVP filters by adding 6 mL lysis buffer [0.73 M sucrose, 40 mM EDTA (pH 8.0), 50 mM Tris (pH 8.3)] to each filter while thawing on ice. Samples were subject to three freeze/thaw cycles using liquid nitrogen and a water bath pre-heated to 40°C. One hundred and twenty microliters of lysozyme solution (5% lysozyme in lysis buffer, 0.2 μm filter sterilized) was added to the filter and the mixture was incubated at 37°C rotating for 45 min. Three hundred microliters of proteinase K solution (1% proteinase K in lysis buffer) and 200 μL SDS (20% solution, 0.2 μm filter sterilized) were added to each filter, and samples were incubated at 55°C rotating for 2 h. Lysate was transferred to 50 mL sterile Teflon centrifuge tubes. Samples were extracted three times using an equal volume to the aqueous phase of phenol:chloroform:isoamyl (25:24:1: pH 8.0) and twice using an equal volume of chloroform-isoamyl alcohol (24:1). Amicon Ultra-15 30k filters were used to wash and concentrate the extracted DNA. Purified samples were washed twice with sterile TE buffer. The pure DNA was stored at -80°C until further analysis. The amount of DNA extracted from the filters was 468, 315, 71, and 52 μg for LVP2, 4, 5, and 8, respectively. Libraries for sequencing were created with Nextera XT (Illumina) reagents following manufacturer’s instructions, except for purification steps, which were done with column cleanup kits (QIAGEN), and library size selection, which was done with BluePippin (Sage Science, Beverly, MA, United States), with a target size of 500 ± 50 bp. Libraries were sequenced with NextSeq 500 (Illumina) in 2 × 150 bp mode using v.1 reagents. The obtained sequence reads were quality-trimmed with Trimmomatic v0.32 (Bolger et al., 2014) using the following settings: -phred33 LEADING:0 TRAILING:5 SLIDINGWINDOW:4:15 MINLEN:36. Paired metagenomic reads were joined using flash version 1.2.11 and the following parameters: -x 0.05 -m 20 -M 150 (Magoč and Salzberg, 2011).

### Transcriptomics of the Crab Spa Diffuse-Flow Vent

Total RNA was extracted from the LVP filters using the same protocol as for the DNA extraction. The only difference in the extraction protocol was that RNA samples were extracted twice with saturated phenol (pH 4.3) and once with chloroform-isoamyl alcohol (24:1). DNA was removed using a TURBO DNA-free kit (AM1907, Ambion) according to manufacturer’s protocol. Samples were screened along with no template controls and a positive *Escherichia coli* control for contaminating bacterial DNA using 8F (5′-AGA GTT TGA TCC TGG CTC AG-3′) and 518R primers (5′-CCGTCAATTCMTTTRAGTTT-3′, IDT) as previously described (Vetriani et al., 1999). DNase-treated RNA (1 μg reactions) was then amplified by *in vitro* transcription using the MessageAmp II aRNA Amplification Kit (Ambion). The aRNA generated was poly-A tailed and precipitated according to the kit instructions using NH_4_OAc and ethanol. RNA (0.2–5 μg) was resuspended in 5 μL DEPC-treated water and reverse-transcribed to double-stranded cDNA using SuperScript Double-Stranded cDNA Synthesis Kit (Invitrogen, Carlsbad, CA, United States). Samples were subsequently stored at -20°C until further analyses.

cDNA was quantified with a Qubit^®^ fluorometer using a dsDNA BR Assay kit (Q32850, Thermo Fisher), and quality was assessed using High Sensitivity D1000 ScreenTape (5067-5584, Agilent) and reagents (5067-5585, Agilent) on an Agilent 2200 TapeStation. Sequencing libraries were prepared using an Ovation Ultralow DR Multiplex System (0331-32, NuGEN) with some modifications to the manufacturer’s protocol. Briefly, cDNA was sheared in a total volume of 130 μl in a microTUBE AFA Fiber Snap-Cap (520045, Covaris) using a Covaris M220 focused-ultrasonicator with the following settings: Peak Incident Power = 50; Duty Factor = 2.0; Cycles per burst = 200; Treatment Time = 160 s. Sheared cDNA was cleaned up using a MinElute Reaction Cleanup kit (28204, QIAGEN). End repair, ligation and amplification steps were performed according to manufacturer’s protocol, but ligation and amplified library purification steps were performed with a QIAquick PCR Purification kit (28104, QIAGEN). Amplified libraries were size-selected on a BluePippin (Sage Sciences) using a 1.5% agarose gel cassette (BDF1510, Sage Sciences) with a size range of 300–500 bp. The size-selected libraries were cleaned up using a MinElute Reaction Cleanup kit, assessed for quality on an Agilent 2200 TapeStation using D1000 ScreenTape and reagents, and quantified with a KAPA Library Quantification Kit (KK4824, KAPA). Libraries were multiplexed and sequenced on an Illumina NextSeq 500 sequencer using a High Output v1 NextSeq kit (FC-404-1004, Illumina).

### Fragment Recruitment of Metagenomic Reads and Characterization of Metagenomic Islands

Metagenomes from the Crab Spa hydrothermal vent were used to search for regions of low fragment recruitment, also known as metagenomic islands, where the metagenomic reads would not go above 75% similarity and would cover less than 50% of the region. BLAST+ v2.2.28 (Camacho et al., 2009) was used to recruit the raw metagenomic reads to each SAG assembly using BLASTn with default parameter values, except for the following: -evalue 0.00001 -soft_masking true -lcase_masking -xdrop_gap 150. Genes present in the metagenomic islands were identified using BLASTx against the GenBank non-redundant (NR) database. The metagenomes were *de novo* assembled using MEGAHIT (Li et al., 2015). VirSorter (Roux et al., 2015) was used to identify viral contigs using both the virome and the RefSeq databases.

To reveal the expressed transcripts of each SAG, fragment recruitment analysis of the paired metatranscriptomic reads against the open reading frame (ORF) was performed, using DIAMOND (Buchfink et al., 2015) at a 95% amino acid identity cut-off. The total counts per ORF were normalized to account for differences in ORF length by calculating the Fragments per ORF Kilobase per Million Mapped fragments (FPKM).

## Results and Discussion

### Identification of Phage-Like Genome Regions

We searched for phage-like DNA sequences in *de novo* assemblies of 126 single amplified genomes (SAGs) of Bacteria and Archaea (Supplementary Table 1) that were obtained from discharged fluids of Crab Spa, a low temperature, diffuse-flow hydrothermal vent. The sequenced SAGs originated from a library of 3,190 SAGs, which was created from both field samples and laboratory experiments, during which the indigenous community was exposed to an elevated temperature and/or amendments of nitrate, oxygen or hydrogen (Sievert and Vetriani, 2012; McNichol et al., 2016, 2018; Supplementary Table 1). A total of 1,176 SAGs could be identified using the 16S rRNA gene. In agreement with prior studies of diverse hydrothermal vents, including Crab Spa (Sievert and Vetriani, 2012; McNichol et al., 2016, 2018), the SAG library was dominated at ∼84% by Campylobacteria, including *Sulfurimonas* (13%), *Arcobacter* (11%), *Sulfurovum* (6%), *Campylobacteraceae* (3%), and *Thioreductor* (2%) (Supplementary Figure 1), which are believed to be predominantly chemoautotrophic primary producers that can use reduced sulfur compounds and/or hydrogen gas as electron donors in combination with nitrate, oxygen and/or sulfur as electron acceptors (Yamamoto and Takai, 2011; Sievert and Vetriani, 2012; Akerman et al., 2013; Vetriani et al., 2014; McNichol et al., 2018).

**FIGURE 1:**
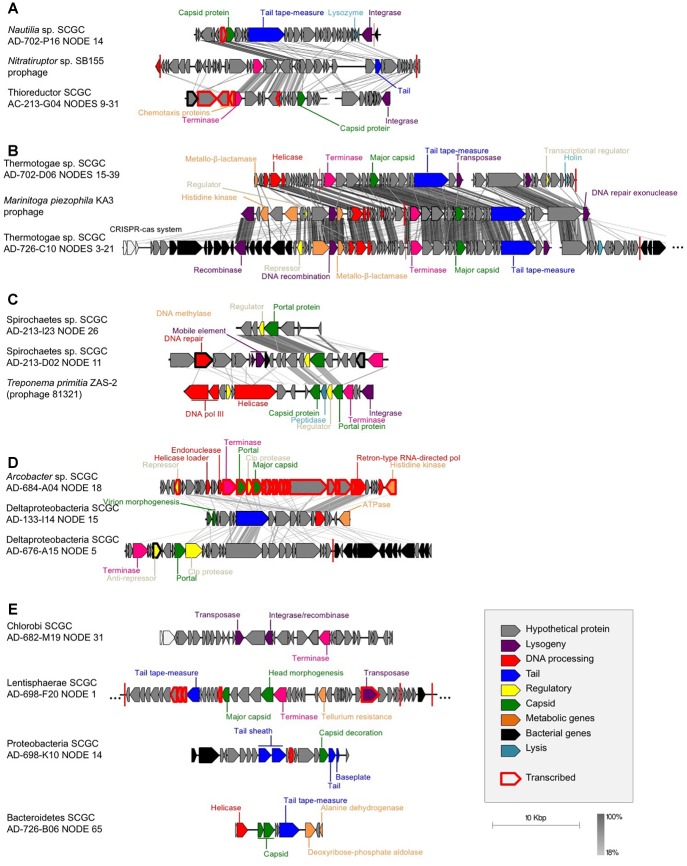
Gene synteny between the identified SAG prophages and their closest relatives from cultured isolates: **(A)** Campylobacteria, **(B)** Thermotogae (*M. piezophila* KA3, accession # NC_016751, positions 301,979-343,554), **(C)** Spirochaetes (*T. primitia* ZAS-2, accession # NC_015578, positions 2,202,498-2,225,178), **(D)** retron-like prophages, and **(E)** prophages with no synteny with previously sequenced phages. tBLASTx was used to identify homologous regions. Each arrow represents a gene, with black arrows representing bacterial genes, showing the putative site of integration. Red vertical bars represent tRNA genes. Gray scale legend indicates DNA sequence identity between genomic sequences. Genes in red bold were found in the metatranscriptomes, which demonstrates their expression in the environment.

Our analysis revealed phage-like genome regions in 13 of the 126 sequenced SAGs (Table 1 and Figure 1). Campylobacteria SAGs AD-702-P16 and AC-213-G04 were found to contain sequences with some homology to a *Siphoviridae* prophage previously identified in *Nitratiruptor* sp. SB155, a campylobacterium that was isolated from an actively venting sulfide mound in the Iheya North hydrothermal field, Japan (Nakagawa et al., 2005, 2007) (Figure 1A). Thermotogae SAGs AD-726-C10 and AD-726-D06 contained sequences related to a *Siphoviridae* prophage previously found in *Marinitoga piezophila*, a bacterium belonging to Thermotogae that was isolated from the Grandbonum deep-sea hydrothermal vent site at 13°N on the East Pacific Rise (Lucas et al., 2012) (Figure 1B). Spirochaetes SAGs AC-213-D02 and AC-213-I23 contained genome regions with homology to a *Siphoviridae* prophage from *Treponema primitia*, a spirochaete previously isolated from termite hindguts (Graber and Breznak, 2004) (Figure 1C). Finally, genome regions from seven phylogenetically diverse SAGs contained retron-like phage sequences (Figure 1D) and sequences with limited similarity in genome organization to previously characterized phages, suggesting the recovery of novel phage types (Figure 1E). It is important to point out that some prophages in the analyzed cells may have remained undetected, due to their insufficient homology to the available reference genomes, and because of the incomplete genome recovery from SAGs (Supplementary Table S1). Moreover, the functionality of the phages cannot be determined given the incompleteness of the recovered prophage genomes.

**Table 1:** SAG-associated viral sequences.

SAG full name	SAG assembly size	Viral contig	Viral contig length	IMG contig ID	Sample source^∗^
*Thioreductor* sp. SCGC AC-213-G04	560,551	NODE_9	17,784	2634579743	*In situ*
		NODE_31	5,905	2634579765	*In situ*
*Nautilia* sp. SCGC AD-702-P16	1,229,711	NODE_14	22,365	2616651450	NO_3_ and H_2_ amendment, 50°C
Thermotogae bacterium SCGC AD-726-C10	1,382,712	NODE_3	51,223	N/A	*In situ*
		NODE_21	17,560	N/A	
Thermotogae bacterium SCGC AD-702-D06	1,610,077	NODE_15	25,047	2616650705	NO_3_ and H_2_ amendment, 50°C
		NODE_39	12,417	2616650729	
Spirochaetes bacterium SCGC AC-213-D02	975,315	NODE_11	26,275	2616648138	*In situ*
Spirochaetes bacterium SCGC AC-213-I23	700,071	NODE_26	10,176	2616648280	*In situ*
*Arcobacter* sp. SCGC AD-684-A04	1,687,778	NODE_18	28,252	2616649007	H_2_ amendment, 24°C
Deltaproteobacteria bacterium SCGC AD-676-A15	1,092,595	NODE_5	36,763	2634580936	*In situ*
Deltaproteobacteria bacterium SCGC AD-133-I14	1,218,259	NODE_15	17,408	2634580581	*In situ*
Chlorobi bacterium SCGC AD-682-M19	669,902	NODE_31	28,515	2634581363	NO_3_ and H_2_ amendment, 24°C
Lentisphaerae bacterium SCGC AD-698-F20	1,000,236	NODE_1	91,859	2634581988	Control, 24°C
Proteobacteria bacterium SCGC AD-698-K10	949,159	NODE_14	22,010	2634582163	Control, 24°C
Bacteroidetes bacterium SCGC AD-726-B06	1,992,123	NODE_65	10,682	2616651820	*In situ*

*^∗^In situ refers to fluid samples retrieved from the natural environment and immediately preserved. Other conditions refer to ∼24 h incubations at in situ pressure as previously described (McNichol et al., 2016).*

### Indications of Lysogeny

**FIGURE 2:**
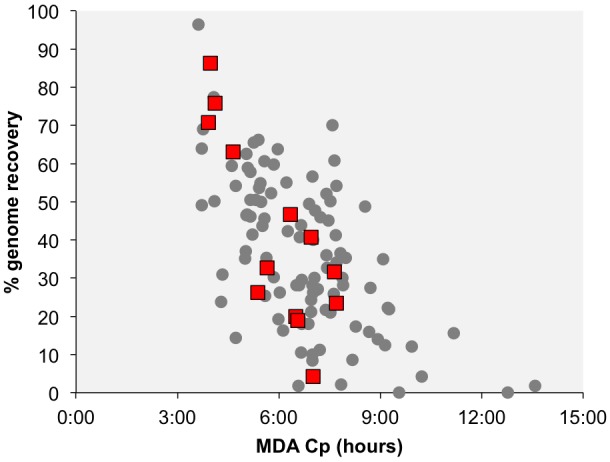
Correlation between the multiple displacement amplification (MDA) critical point (Cp) and the estimated genome recovery. Each data point represents a SAG, with the red squares indicating SAGs that contain phage-like sequences. The Cp is the time required to reach half of the maximal fluorescence of DNA labeled with SYTO-9 stain during single cell MDA reactions.

Many of the identified phage-like regions in SAGs were related to previously described prophages, indicating that they may constitute temperate phages. Two contigs containing phage-like sequences, Deltaproteobacteria AD-676-A15_NODE_5 and Thermotogae bacterium AD-726-C10_NODE_3, encoded both viral and host genes, providing direct evidence for the viral integration into the host’s chromosome (Table 1 and Figure 1). Additionally, phage-like genomic regions in SAGs Lentisphaerae AD-698-F20 and *Nautilia* sp. AD-702-P16 were flanked by tRNA genes, which are common sites of prophage integration (Lowe and Eddy, 1996). Furthermore, phage-like genomic regions encoded recombinases in SAGs Chlorobi AD-682-M19 and Thermotogae AD-726-C10, integrases in *Nautilia* sp. AD-702-P16, and transposases in five SAGs (Spirochaetes AD-213-D02, Thermotogae AD-702-D06, Thermotogae AD-726-C10, Chlorobi AD-682-M19, and Lentisphaerae AD-698-F20), providing additional indications for potential lysogeny (Table 1 and Figure 1). The *de novo* assembly of prophage integration sites from short fragment reads is notoriously difficult, due to their frequent co-location with repeat regions (Zerbino and Birney, 2008), and is further impaired by the incomplete genome recovery from single cells (Labonté et al., 2015a). Thus, the relationship between the kinetics of single cell MDA reaction and the recovery of host genome has been employed as a complementary source of evidence when discriminating lytic infections and lysogens in single cell genomics studies (Labonté et al., 2015b). Typically, a negative correlation between the MDA critical point (MDA Cp) and the genome recovery is observed with SAGs originating from cells that undergo lytic infections, forming outliers in this relationship. This is due to the infection-induced degradation of host genome while maintaining high, phage-dominated content of total DNA in a cell (Labonté et al., 2015b). In this study, none of the SAGs with phage-like sequences formed obvious outliers from this correlative pattern as it was observed in surface oceanic samples where lytic infections were more prevalent (Figure 2). The fact that phage sequences share homology with other prophages, the observation of complete prophages within a contig with flanked host sequences, and the MDA Cp kinetics, all suggest that most if not all of the detected phage-like genomic regions represent prophages. Thus, multiple lines of evidence suggest that the identified phages are temperate phages, or prophages.

Several other reports that rely on different research tools also suggest high prevalence of prophages in hydrothermal systems, as compared to the overlaying water column (Williamson et al., 2008; Anderson et al., 2011a). Likewise, our single cell genomics analysis of *Desulforudis audaxviator* revealed an abundance of lysogens and no evidence for lytic infections in this lineage that inhabits deep, terrestrial subsurface environment (Labonté et al., 2015a). In contrast, the application of identical methodology to sort and amplify the cells, along with the same analytical tools on surface ocean bacterioplankton revealed lytic infections in a third of the analyzed SAGs while providing no indications of lysogens (Labonté et al., 2015b). This is in good agreement with our current understanding of the frequency and types of phage infections in marine bacterioplankton (Weinbauer et al., 2003; Paul, 2008). To confirm the absence of lytic infections, we searched the Crab Spa metagenomic data (LPV2 and LVP4) to identify viral contigs using VirSorter (Roux et al., 2015). Using the virome database, no viral contig was identified. Using the RefSeq database, we found no viral contig in LPV4 (out of 103,028 contigs) and two small contigs (466 and 831 bp) in LPV2 (57,013). The absence of a strong viral signal can also point to the absence of lytic infections.

One hypothesis postulates that lysogeny is more prevalent than lytic infections in starving cells, which may not permit efficient production of phage particles (Wommack and Colwell, 2000; Weinbauer, 2004; Paul, 2008). This is, however, unlikely in the Crab Spa hydrothermal system where Campylobacteria actively divide, fix carbon, and produce biomass (McNichol et al., 2018). Another hypothesis suggests that lysogeny is a response to changes in the cell’s environment and physiology (Maurice et al., 2013), which appears relevant to deep-sea hydrothermal vents where the mixing of hydrothermal fluid with seawater creates rapidly changing conditions. However, this hypothesis is also unlikely in this particular case as the cells sampled here are adapted to these conditions and were actively growing in this mixing zone below the seafloor, most likely by forming biofilms, exploiting the available energy created by the mixing of reduced and oxidized fluids to perform chemosynthesis.

Campylobacteria represented ∼90% of the SAG library, yet very few infections were observed. Since Campylobacteria were highly active (McNichol et al., 2018), a lytic infection at the time of sampling could lead to cell lysis at the time of cell preservation aboard the research vessel for subsequent single cell sorting. Perhaps the active growth of Campylobacteria explains why phages in a state of lytic infection were not found in SAGs. Infection by a lytic phage is fast under optimal conditions but could proceed slowly in starving cells. The low frequency of infected cells may reduce the probability of sorting infected cells. This could explain why no lytic infections were observed in the cells.

CRISPR/Cas systems have been shown to be prevalent in diffuse-flow hydrothermal system, likely providing resistance to phage infections. In one study, they were even able to associate phages to their host using CRISPR spacer sequence matches in metagenomic data (Anderson et al., 2011b). As many as 55 of the 126 (44%) analyzed SAGs, including 36 (29% of total) Campylobacteria (Supplementary Table 1), contained CRISPR/Cas, the immune system of bacteria and archaea (Boyaval et al., 2007). The prevalence of CRISPR/Cas in the Crab Spa vent may be yet another factor contributing to the resistance of these cells to phage infections.

**FIGURE 3:**
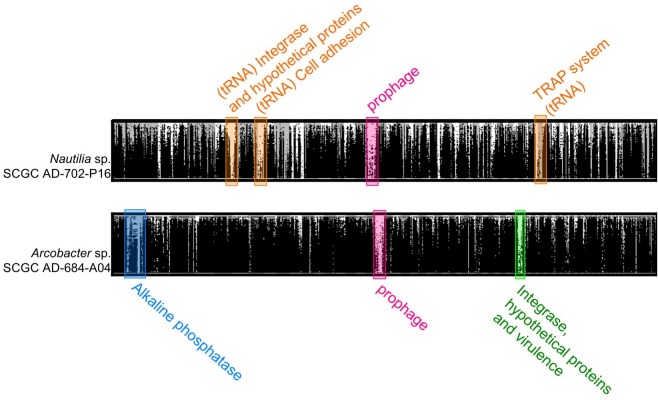
Fragment recruitment plot of metagenomic reads from Crab Spa and Teddy Bear hydrothermal vents on the contigs from SAGs *Nautilia* sp. AD-702-P16 and *Arcobacter* AD-684-A04. Metagenomic recruitment was performed using BLASTx and hits with more than 50% identity are displayed.

In order to determine how conserved the putative prophages are in diffuse-flow hydrothermal vents of the study area, we used the prophage-containing SAGs as references to recruit fragments of two metagenomes from the Crab Spa vent and two metagenomes from a nearby vent named Teddy Bear. We found prophages to be poor metagenome recruiters, as compared to other genome regions (Figure 3). This suggests that the identified prophages are present only in a small fraction of related cells that inhabit the studied hydrothermal vents. Such regions of low metagenomic recruitment are known as metagenomic islands (Rodriguez-Valera et al., 2009) and usually consist of mobile DNA elements and prophages. In the campylobacterial SAGs AD-684-A04 and AD-702-P16, which were highly represented in the metagenomes, additional metagenomic islands encoded for a cell adhesion protein, a Tripartite ATP-independent periplasmic (TRAP) transporter system, an alkaline phosphatase, an integrase, and genes involved in virulence. Products of some of these genes are potential phage recognition sites as they are genes expressed at the surface of the cells, suggesting a role in phage evasion. Previous studies also observed enrichment in phage recognition sites in metagenomic islands in marine genomes, suggesting strain-specificity in phage-interacting genes (Rodriguez-Valera et al., 2009). Here, we show that prophages and infection-related genes are a predominant type of metagenomic island in the studied hydrothermal vent.

### Potential Impact of Identified Prophages on Their Hosts

Metatranscriptomic analysis of the Crab Spa fluid microbiome demonstrated that multiple prophage-encoded genes are transcribed in this environment (Figure 1; genes in bold red). The prophage of the *Arcobacter* SAG AD-684-A04 was fully transcribed, suggesting that this prophage was inducing lytic infections in some members of the vent microbial community. Four other prophages also contained some transcribed genes. Among them were transposases and integrases, suggesting that phage-mediated horizontal gene transfer and recombination are contributing to microbial adaptation and evolution. Several Crab Spa prophages contained genes for metabolic features that may benefit their hosts. It is common for prophages to express one gene or small gene cassettes when in a dormant state. These genes, referred to as “morons” may contribute to host fitness. Some genes identified in the prophages could provide a fitness advantage to their host. In one example, a prophage in the *Thioreductor* sp. SAG AC-213-G04 encodes two genes involved in energy chemotaxis (Accession numbers ACM93792 and ACM93615; Supplementary Table 2), potentially allowing for the movement of an organism toward an energy source, as previously reported for a prophage of a *Pseudoalteromonas* strain isolated from Arctic sea ice (Yu et al., 2014). In another example, a prophage in Lentisphaerae SAG AD-698-F20 encodes a putative tellurium resistance gene (Accession number KKT02083 and RAST annotations; Supplementary Table 2). Tellurium is a toxic element that is present in relatively high concentrations in some hydrothermal vents (Csotonyi et al., 2006). Therefore, tellurium resistance may provide a significant advantage for a vent bacterium. While many strains resistant to metalloids have been isolated from hydrothermal vents (Rathgeber et al., 2002), this may be the first report of a tellurium resistance gene on a prophage. Yet another example is putative metallo-beta-lactamase genes on prophages of Thermotogae SAGs AD-702-D06 (accession number WP_014295825) and AD-726-C10 (accession number WP_003366035), which may be involved in a multi-drug resistance of the host (Palzkill, 2013). It is plausible that additional host-relevant features are encoded by the many hypothetical proteins on the Crab Spa prophages, the function of which remains unknown. Collectively, these data suggest complex and active roles of prophages in the Crab Spa microbial community.

**FIGURE 4:**
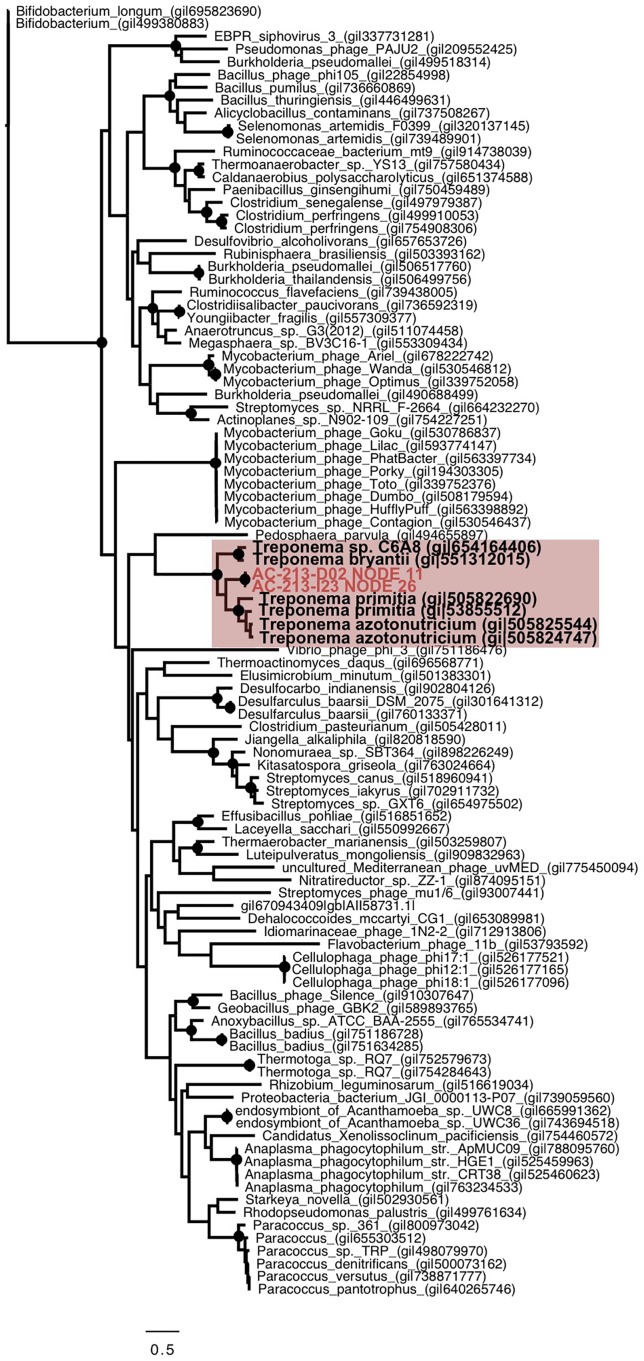
Phylogenetic analysis of the portal protein from Spirochaetes SAGs (red), Spirochaetes isolates (bold) and other bacteria and phages, showing segregation of the sequences based on their host. The tree was generated using maximum likelihood, with 100 bootstrap replicates, using the LG model with a gamma distribution (+G), estimated rates of variation among sites and a proportion of invariable sites (+I). >99% bootstrap support is indicated as black circles at nodes. Scale bar represents the number of amino acid substitution per site.

**FIGURE 5:**
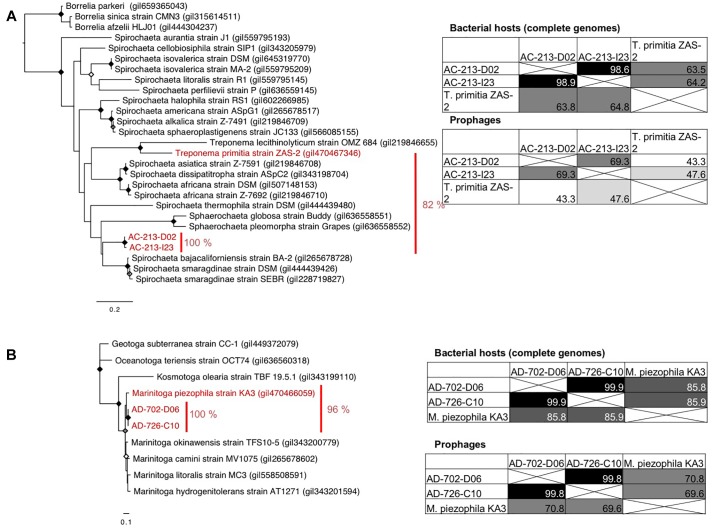
Similarity of the SSU rRNA genes (phylogenetic trees), host genomes (average nucleotide identity), and the corresponding prophage genomes (overall similarity) of Spirochaetes **(A)** and Thermotogae **(B)**. Maximum likelihood phylogenetic trees were generated using the GTR model with a gamma distribution (+G), estimated rates of variation among sites, and a proportion of invariable sites (+I). Bootstrap support by 100, ≥85, and ≥75% replicates out of 100 are shown by black, gray, and white dots at the nodes.

We observed consistency in genome organization and host phylogeny among prophages retrieved from Spirochaetes and Thermatogae. There is partial genome synteny and the monophyly of portal proteins of prophages in Spirochaetes SAGs from hydrothermal vents and the *T. primitia* strain ZAS-2 from termite guts (Figure 1, 4). This implies phage–host co-evolution that predates the last common ancestor of Spirochaetes residing in these distant habitats. Considering that the two Spirochaetes SAGs and *T. primitia* share only 82% identity in their SSU rRNA gene, and assuming that 1% divergence of the 16S rRNA gene takes an average of 50 million years (Ochman and Wilson, 1987), it is plausible that the last common ancestor of these cells lived around one billion years ago (Figure 5). The higher average nucleotide similarity among hosts (63–64%) than the conserved regions of prophages (43–47%) shows that the divergence of these prophages outpaced the divergence of their hosts. The same is true for Thermotogae, where the average nucleotide similarity between SAGs AD-702-D06 and AD-726-C10 on the one hand and the *M. piezophila* strain KA3 on the other hand (∼85%) was greater than the nucleotide similarity of the shared genes of their prophages (∼70%) (Figure 5). Notably, all three Thermotogae originate from hydrothermal vents on the East-Pacific Rise and there is a 96% identity in the SSU rRNA gene sequence between SAGs and the isolate, suggesting a more recent common ancestor, as compared to Spirochaetes. These examples of prophage–host co-evolution over geological time scales provide further support for strong co-dependence of the detected prophages and their hosts, but also that the mutation rate and selection pressure differ between prophages and their hosts.

## Conclusion

Our results suggest the predominance of lysogenic over lytic phage–host interaction in a diffuse-flow, deep-sea hydrothermal vent. This is in line with prior findings from other hydrothermal vents and contrasts with the prevalence of lytic infections in surface ocean bacterioplankton, likely reflecting differences in the stability of these two ecosystems. Resistance to phage infection in the dominant members of the community such as Campylobacteria could be due to a high prevalence of CRISPR regions. We show that some of the identified prophages have co-evolved with their host over geological time scales and that their genes are transcribed in the environment. Functional annotations of lysogeny genes suggest involvement in horizontal gene transfer and in protection of the host against toxic metals and anti-bacterial compounds.

## Data Availability

Viral contig sequences, phylogenetic trees and alignments are available in Supplementary Material of this publication. SAG genomic sequences are available on the D.O.E. Joint Genome Institutes IMG portal, https://img.jgi.doe.gov (Supplementary Table 1 for accession numbers). Metagenomes LVP2 and LVP4 and metatranscriptomes CV88 and LPV4 are available on the MG-RAST server under the accession numbers mgm4679649.3, mgm4679741.3, mgm4773053.3, and mgm4773182.3, respectively.

## Author Contributions

JL performed the phage identification and analysis, prepared the figures, and wrote the manuscript. MP analyzed the SAG genomes, performed the metagenomic and transcriptomic fragment recruitment, and participated in the manuscript preparation. SMS performed the SAG sampling in 2008 and the LVP sampling. EF generated the SAGs used in this study. JM performed the incubations from which SAGs were generated and participated in the manuscript preparation. AG and LG performed the DNA and RNA extractions for the metagenomics and transcriptomics analyses, and participated in the manuscript preparation. CV, SMS, and RS designed the experiments and participated in the manuscript preparation. RS lead the generation of the SAGs, helped in data analysis and participated in the manuscript preparation.

## Conflict of Interest Statement

The authors declare that the research was conducted in the absence of any commercial or financial relationships that could be construed as a potential conflict of interest.

## Footnotes

1 scgc.bigelow.org
