# Supplementary material for: Single Cell Genomics-Based Analysis of Gene Content and Expression of Prophages in a Diffuse-Flow Deep-Sea Hydrothermal System
Source: Front Microbiol. 2019 Jun 12;10:1262. doi: 10.3389/fmicb.2019.01262 (PMC6581674; doi:10.3389/fmicb.2019.01262)
Supplement: Supplementary file 2 [file Data_Sheet_2.docx]

**SUPPLEMENTARY MATERIAL**


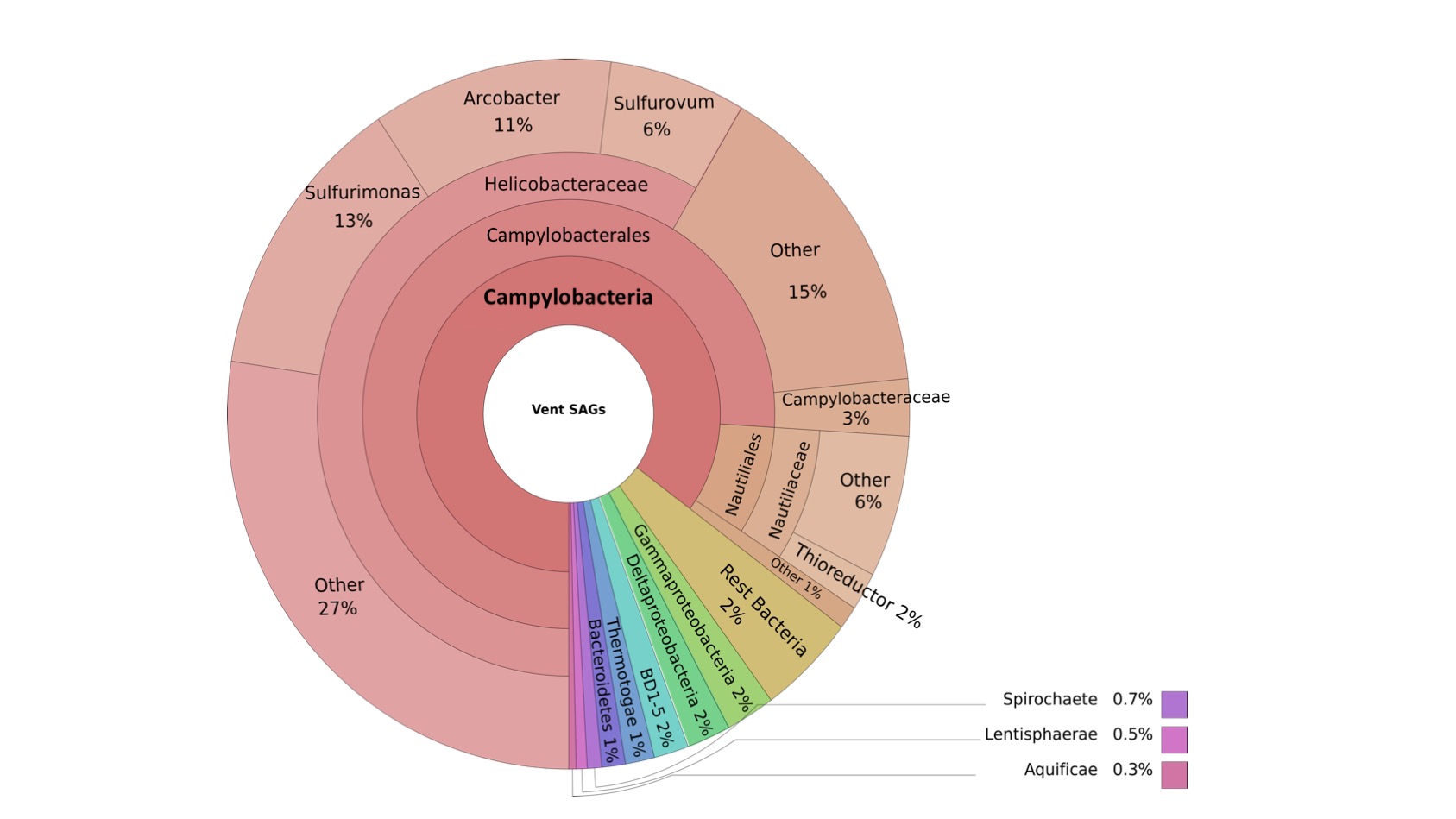


**Supplementary Figure 1:** Phylogenetic composition of the 1,176 identified SAGs based on 16S rRNA.

**Supplementary Table 1:** SAG sequencing and assembly characteristics.

**In situ* refers to fluid samples retrieved from the natural environment and immediately preserved. Other conditions refer to ~24 h incubations at *in situ* pressure as previously described (McNichol et al., 2016).

| **Public ID** | **JGI taxon ID** | **Sample Source*** | **Assembly length** | **Max. contig length** | **Contig count** | **GC (%)** | **Estimated genome completeness (%)** | **Sample collection date** | **CRISPR count** |
| --- | --- | --- | --- | --- | --- | --- | --- | --- | --- |
| *Aquificae* bacterium SCGC AAA036-B04 | 2616644856 | In situ (2008) | 480,251 | 41,646 | 39 | 39 | 25.9 | 10/1/08 | 0 |
| *Sulfurovum sp.* SCGC AAA036-I20 | 2616644857 | In situ (2008) | 1,022,980 | 53,915 | 94 | 34 | 54.3 | 10/1/08 | 0 |
| *Arcobacter sp.* SCGC AAA036-J15 | 2616644858 | In situ  (2008) | 893,823 | 51,444 | 96 | 27 | 43.1 | 10/1/08 | 2 |
| *Sulfurovum sp.* SCGC AAA036-J22 | 2616644859 | In situ  (2008) | 410,627 | 41,213 | 42 | 33 | 24.1 | 10/1/08 | 3 |
| *Sulfurovum sp.* SCGC AAA036-N17 | 2616644860 | In situ  (2008) | 469,609 | 53,826 | 33 | 34 | 15.5 | 10/1/08 | 2 |
| *Sulfurovum sp.* SCGC AAA036-P16 | 2616644861 | In situ  (2008) | 179,730 | 10,181 | 36 | 34 | 2.4 | 10/1/08 | 1 |
| *Aquificae* bacterium SCGC AAA040-G05 | 2616644862 | In situ  (2008) | 432,678 | 32,456 | 47 | 40 | 14.0 | 10/1/08 | 4 |
| *Caminibacter* SCGC AC-211-B08 | 2634166780 | In situ  (2008) | 884,994 | 74,786 | 61 | 34 | 52.1 | 10/1/08 | 2 |
| *Nautilia* SCGC AC-211-P17 | 2634166781 | In situ  (2008) | 1,092,418 | 83,160 | 71 | 34 | 70.1 | 10/1/08 | 0 |
| *Sulfurovum sp.* SCGC AC-213-A03 | 2616644863 | In situ  (2008) | 430,374 | 40,619 | 49 | 34 | 25.8 | 10/1/08 | 1 |
| Archaeon SCGC AC-213-A17 |  | In situ  (2008) | 0 | 0 | 0 | 0 | N/A | 10/1/08 | 0 |
| Sulfurovum SCGC AC-213-A20 | 2634166829 | In situ  (2008) | 469,766 | 40,335 | 37 | 33 | 21.7 | 10/1/08 | 0 |
| *Arcobacter* SCGC AC-213-B06 | 2634166830 | In situ  (2008) | 734,870 | 46,059 | 64 | 28 | 35.4 | 10/1/08 | 1 |
| Bacterium SCGC AC-213-C05 | 2616644864 | In situ  (2008) | 661,067 | 41,419 | 70 | 31 | 21.1 | 10/1/08 | 0 |
| *Spirochaetes sp.*  SCGC AC-213-D02 | 2616644865 | In situ  (2008) | 975,315 | 70,366 | 78 | 36 | 31.7 | 10/1/08 | 0 |
| Archaeon SCGC AC-213-D04 | 2634166831 | In situ  (2008) | 633,463 | 46,808 | 58 | 34 | 28.1 | 10/1/08 | 2 |
| *Sulfurovum sp.* SCGC AC-213-D23 | 2616644866 | In situ  (2008) | 479,203 | 38,108 | 49 | 33 | 24.3 | 10/1/08 | 2 |
| Sulfurovum SCGC AC-213-E03 | 2634166832 | In situ  (2008) | 856,926 | 84,459 | 75 | 43 | 36.6 | 10/1/08 | 0 |
| Archaeon SCGC AC-213-E04 | 2634166833 | In situ  (2008) | 291,636 | 65,113 | 16 | 28 | 18.0 | 10/1/08 | 1 |
| Bacterium SCGC AC-213-F14 | 2634166834 | In situ  (2008) | 1,015,903 | 85,374 | 95 | 31 | 34.0 | 10/1/08 | 2 |
| Campylobacteria bacterium SCGC AC-213-F18 (deposited as Epsilonproteobacteria bacterium SCGC AC-213-F18) | 2634166835 | In situ  (2008) | 839,367 | 58,736 | 76 | 36 | 30.1 | 10/1/08 | 0 |
| Thioreductor SCGC AC-213-G04 | 2634166836 | In situ  (2008) | 560,551 | 37,647 | 65 | 27 | 26.2 | 10/1/08 | 0 |
| *Sulfurimonas* SCGC AC-213-G11 | 2634166837 | In situ  (2008) | 519,684 | 38,494 | 46 | 33 | 26.2 | 10/1/08 | 2 |
| Spirochaetes sp. SCGC AC-213-!02 (deposited as Bacterium SCGC AC-213-I02) | 2634166838 | In situ  (2008) | 939,320 | 116,375 | 84 | 35 | 30.1 | 10/1/08 | 0 |
| *Spirochaetes sp.*  SCGC AC-213-I23 | 2616644867 | In situ  (2008) | 700,071 | 57,887 | 49 | 37 | 4.2 | 10/1/08 | 0 |
| Bacterium SCGC AC-213-J07 | 2634166839 | In situ  (2008) | 460,862 | 26,119 | 49 | 30 | 15.9 | 10/1/08 | 0 |
| Bacterium SCGC AC-213-K14 | 2634166840 | In situ  (2008) | 548,207 | 66,982 | 33 | 29 | 34.9 | 10/1/08 | 0 |
| Bacterium SCGC AC-213-K20 | 2616644868 | In situ  (2008) | 884,938 | 98,660 | 65 | 43 | 22.3 | 10/1/08 | 0 |
| Bacterium SCGC AC-213-L19 | 2634166841 | In situ  (2008) | 154,133 | 32,001 | 14 | 31 | 17.2 | 10/1/08 | 0 |
| Archaeon SCGC AC-213-M03 | 2634166842 | In situ  (2008) | 546,745 | 36,415 | 76 | 23 | 46.0 | 10/1/08 | 0 |
| Bacterium SCGC AC-213-N07 | 2634166843 | In situ  (2008) | 459,246 | 38,736 | 62 | 27 | 21.9 | 10/1/08 | 0 |
| Bacterium SCGC AC-213-N20 | 2616644869 | In situ  (2008) | 1,080,196 | 92,826 | 64 | 41 | 47.8 | 10/1/08 | 3 |
| Archaeon SCGC AC-213-O17 | 2634166845 | In situ  (2008) | 434,337 | 57,032 | 29 | 30 | 27.0 | 10/1/08 | 0 |
| *Sulfurovum sp.* SCGC AC-213-P02 | 2616644870 | In situ  (2008) | 882,222 | 51,286 | 68 | 33 | 43.9 | 10/1/08 | 3 |
| *Sulfurovum sp.* SCGC AD-133-C09 | 2616644871 | In situ  (2008) | 480,720 | 73,916 | 27 | 45 | 27.5 | 10/1/08 | 0 |
| *Sulfurovum sp.* SCGC AD-133-F15 | 2616644872 | In situ  (2008) | 805,653 | 71,936 | 79 | 34 | 40.0 | 10/1/08 | 2 |
| Campylobacter SCGC AD-133-F21 (deposited as Campylobacteriales bacterium SCGC AD-133-F21) | 2616644873 | In situ  (2008) | 365,423 | 49,299 | 24 | 44 | 27.1 | 10/1/08 | 0 |
| *Sulfurovum sp.* SCGC AD-133-G07 | 2616644874 | In situ  (2008) | 229,121 | 15,040 | 38 | 33 | 15.5 | 10/1/08 | 1 |
| *Sulfurovum sp.* SCGC AD-133-G19 | 2616644875 | In situ  (2008) | 259,558 | 22,014 | 32 | 56 | N/A | 10/1/08 | 0 |
| *Aquificae* bacterium SCGC AD-133-I03 | 2616644876 | In situ  (2008) | 129,820 | 12,859 | 28 | 33 | 18.0 | 10/1/08 | 0 |
| Bacterium SCGC AD-133-I14 | 2634166852 | In situ  (2008) | 1,218,259 | 35,122 | 148 | 30 | 40.8 | 10/1/08 | 0 |
| Bacterium SCGC AD-133-K22 | 2634166853 | In situ  (2008) | 1,339,044 | 136,346 | 108 | 32 | 35.9 | 10/1/08 | 0 |
| *Sulfurovum sp.* SCGC AD-133-N19 | 2616644877 | In situ  (2008) | 1,185,022 | 92,478 | 95 | 34 | 60.9 | 10/1/08 | 6 |
| Bacterium SCGC AD-133-O04 | 2634166854 | In situ  (2008) | 760,047 | 83,473 | 39 | 49 | 50.2 | 10/1/08 | 0 |
| Bacterium SCGC AD-676-A15 | 2634166859 | NO_3_ and H_2_ amendment, 50ºC | 1,092,595 | 154,144 | 90 | 29 | 32.8 | 1/15/14 | 0 |
| Archaeon SCGC AD-676-D11 | 2634166860 | NO_3_ and H_2_ amendment, 50ºC | 1,102,100 | 35,337 | 135 | 48 | 49.4 | 1/15/14 | 9 |
| Bacterium SCGC AD-676-N04 | 2634166861 | NO_3_ and H_2_ amendment, 50ºC | 851,680 | 47,819 | 90 | 32 | 21.3 | 1/15/14 | 0 |
| *Sulfurovum sp.* SCGC AD-682-E17 | 2616644878 | NO_3_ and H_2_ amendment, 24ºC | 596,468 | 37,867 | 65 | 31 | N/A | 1/15/14 | 2 |
| Archaeon SCGC AD-682-J14 | 2634166863 | NO_3_ and H_2_ amendment, 24ºC | 326,734 | 76,125 | 30 | 43 | 14.0 | 1/15/14 | 0 |
| Chlorobi SCGC AD-682-M19  (deposited as Bacterium SCGC AD-682-M19) | 2634166864 | NO_3_ and H_2_ amendment, 24ºC | 2,625,066 | 92,134 | 129 | 34 | 63.1 | 1/15/14 | 0 |
| *Sulfurimonas* sp. SCGC AD-682-M21 | 2616644879 | NO_3_ and H_2_ amendment, 24ºC | 1,022,834 | 48,148 | 91 | 31 | 59.3 | 1/15/14 | 2 |
| Alphaproteobacteria bacterium SCGC AD-682-O19 | 2616644880 | NO_3_ and H_2_ amendment, 24ºC | 109,007 | 34,869 | 10 | 58 | 1.7 | 1/15/14 | 0 |
| *Arcobacter sp.* SCGC AD-684-A04 | 2616644881 | H_2_ amendment | 1,687,778 | 109,550 | 107 | 26 | 86.2 | 1/15/14 | 3 |
| *Thioreductor* sp. SCGC AD-684-A05 | 2616644882 | H_2_ amendment | 1,849,279 | 134,134 | 72 | 26 | 96.3 | 1/15/14 | 3 |
| *Thioreductor* sp. SCGC AD-684-B23 | 2616644883 | H_2_ amendment | 1,090,123 | 37,157 | 132 | 27 | 63.9 | 1/15/14 | 0 |
| Archaeon SCGC AD-684-D18 | 2634166865 | H_2_ amendment | 532,869 | 116,175 | 32 | 35 | 53.7 | 1/15/14 | 0 |
| *Arcobacter sp.* SCGC AD-684-E06 | 2616644884 | H_2_ amendment | 272,965 | 40,108 | 22 | 28 | 14.4 | 1/15/14 | 1 |
| Bacterium SCGC AD-684-G02 | 2634166866 | H_2_ amendment | 80,328 | 35,441 | 3 | 34 | 1.8 | 1/15/14 | 0 |
| Bacterium SCGC AD-684-I09 | 2634166867 | H_2_ amendment | 864,690 | 52,271 | 69 | 44 | 16.3 | 1/15/14 | 2 |
| *Sulfurimonas* sp. SCGC AD-684-O18 | 2616644885 | H_2_ amendment | 1,506,914 | 78,177 | 120 | 30 | 70.4 | 1/15/14 | 1 |
| Bacterium SCGC AD-687-F17 | 2616644886 | NO_3_ amendment | 1,865,503 | 79,940 | 155 | 38 | 55.0 | 1/15/14 | 0 |
| Thaumarchaeon SCGC AD-687-J09 | 2616644887 | NO_3_ amendment | 409,841 | 35,948 | 36 | 45 | 41.2 | 1/15/14 | 0 |
| *Halomonas* sp. SCGC AD-687-O20 | 2616644888 | NO_3_ amendment | 620,301 | 147,776 | 45 | 55 | 12.1 | 1/15/14 | 0 |
| Bacterium SCGC AD-687-P10 | 2634166868 | NO_3_ amendment | 2,471,740 | 95,579 | 205 | 42 | 60.7 | 1/15/14 | 0 |
| *Sulfurimonas* sp. SCGC AD-690-A20 | 2616644889 | O_2_ amendment | 1,189,252 | 61,798 | 88 | 36 | 54.1 | 1/15/14 | 0 |
| *Sulfurimonas* sp. SCGC AD-690-C15 | 2616644890 | O_2_ amendment | 718,737 | 113,529 | 30 | 31 | 30.9 | 1/15/14 | 0 |
| Bacterium SCGC AD-690-K15 |  | O_2_ amendment | 546,629 | 49,732 | 54 | 42 | 8.4 | 1/15/14 | 0 |
| *Marinobacter* sp. SCGC AD-690-L09 | 2616644891 | O_2_ amendment | 216,841 | 116,958 | 11 | 57 | 4.2 | 1/15/14 | 0 |
| *Sulfurimonas* sp. SCGC AD-690-P11 | 2616644892 | O_2_ amendment | 570,211 | 62,419 | 33 | 34 | 23.7 | 1/15/14 | 0 |
| Epsilonproteobacteria SCGC AD-698-A03 | 2616644893 | Control | 378,843 | 35,464 | 33 | 42 | 30.3 | 1/15/14 | 0 |
| Campylobacter SCGC AD-698-A20 (deposited as Campylobacteriales bacterium SCGC AD-698-A20) | 2616644894 | Control | 662,812 | 41,609 | 57 | 34 | 35.3 | 1/15/14 | 2 |
| Bacterium SCGC AD-698-B07 | 2634166870 | Control | 798,648 | 65,553 | 67 | 26 | 52.3 | 1/15/14 | 0 |
| Bacteroidetes bacterium SCGC AD-698-B15 | 2616644895 | Control | 1,812,196 | 60,345 | 182 | 28 | 63.8 | 1/15/14 | 1 |
| *Sulfurovum sp.* SCGC AD-698-D13 | 2616644896 | Control | 502,990 | 41,870 | 56 | 34 | 19.3 | 1/15/14 | 1 |
| Bacterium SCGC AD-698-E03 | 2634166871 | Control | 1,204,332 | 55,509 | 96 | 31 | 25.4 | 1/15/14 | 2 |
| Bacterium SCGC AD-698-F20 | 2634166872 | Control | 1,000,236 | 91,859 | 110 | 42 | 19.0 | 1/15/14 | 1 |
| *Sulfurovum sp.* SCGC AD-698-G16 | 2616644897 | Control | 1,015,564 | 72,437 | 75 | 34 | 56.6 | 1/15/14 | 0 |
| Microgenomates bacterium SCGC AD-698-G18 | 2634166873 | Control | 404,538 | 29,893 | 52 | 34 | 28.1 | 1/15/14 | 0 |
| Gracilibacteria bacterium SCGC AD-698-G19 | 2636415964 | Control | 305,480 | 13,940 | 75 | 23 | 19.3 | 1/15/14 | 0 |
| Bacterium SCGC AD-698-I09 | 2616644898 | Control | 414,175 | 24,143 | 57 | 43 | 2.2 | 1/15/14 | 0 |
| *Sulfurovum sp.* SCGC AD-698-J17 | 2616644899 | Control | 645,254 | 29,877 | 90 | 33 | 28.2 | 1/15/14 | 1 |
| Bacterium SCGC AD-698-K10 | 2634166874 | Control | 949,159 | 35,676 | 111 | 47 | 23.5 | 1/15/14 | 0 |
| Archaeon SCGC AD-698-L20 | 2634166875 | Control | 456,820 | 67,647 | 31 | 32 | 35.1 | 1/15/14 | 2 |
| Alphaproteobacteria bacterium SCGC AD-698-M03 | 2616644900 | Control | 67,472 | 36,799 | 7 | 58 | 0.0 | 1/15/14 | 0 |
| Bacterium SCGC AD-698-M19 | 2634166876 | Control | 669,902 | 34,393 | 83 | 30 | 26.2 | 1/15/14 | 0 |
| Omnitrophica bacterium SCGC AD-698-N15 | 2634166877 | Control | 949,576 | 75,300 | 62 | 37 | 37.1 | 1/15/14 | 0 |
| Gracilibacteria bacterium SCGC AD-698-O07 | 2636415965 | Control | 1,170,098 | 44,634 | 190 | 26 | 65.5 | 1/15/14 | 1 |
| Bacterium SCGC AD-698-P15 | 2634166878 | Control | 644,396 | 53,041 | 55 | 47 | 11.2 | 1/15/14 | 0 |
| Bacterium SCGC AD-699-J03 | 2634166879 |  | 1,313,496 | 79,483 | 99 | 47 | 48.8 | 1/15/14 | 0 |
| *Thioreductor* sp. SCGC AD-699-O04 | 2616644901 | O_2_ amendment | 1,352,300 | 49,217 | 162 | 26 | 77.3 | 1/15/14 | 0 |
| *Thioreductor* sp. SCGC AD-702-C13 | 2616644902 | NO_3_ and H_2_ amendment, 50ºC | 960,946 | 35,833 | 131 | 26 | 50.2 | 1/15/14 | 2 |
| *Thermotogae* bacterium SCGC AD-702-D06 | 2616644903 | NO_3_ and H_2_ amendment, 50ºC | 1,610,077 | 113,099 | 127 | 29 | 70.7 | 1/15/14 | 4 |
| *Marinobacter* sp. SCGC AD-702-E19 | 2616644904 | NO_3_ and H_2_ amendment, 50ºC | 56,805 | 25,478 | 5 | 54 | 0.0 | 1/15/14 | 0 |
| *Nautilia* sp. SCGC AD-702-J15 | 2616644905 | NO_3_ and H_2_ amendment, 50ºC | 1,027,675 | 40,392 | 119 | 30 | 49.1 | 1/15/14 | 1 |
| Campylobacteria bacterium SCGC AD-702-K02  (Deposited as “Epsilonproteobacteria bacterium ACGC AD-702-K02) | 2616644906 | NO_3_ and H_2_ amendment, 50ºC | 539,528 | 43,185 | 38 | 42 | 43.6 | 1/15/14 | 0 |
| *Sulfurovum sp.* SCGC AD-702-L15 | 2616644907 | NO_3_ and H_2_ amendment, 50ºC | 1,026,723 | 73,535 | 82 | 34 | 54.9 | 1/15/14 | 5 |
| Bacteroidetes bacterium SCGC AD-702-M03 | 2616644908 | NO_3_ and H_2_ amendment, 50ºC | 1,425,649 | 87,348 | 110 | 40 | 41.4 | 1/15/14 | 2 |
| *Thermotogae* bacterium SCGC AD-702-M05 | 2616644909 | NO_3_ and H_2_ amendment, 50ºC | 1,648,320 | 72,348 | 146 | 29 | 69.0 | 1/15/14 | 1 |
| *Thioreductor* sp. SCGC AD-702-O18 | 2616644910 | NO_3_ and H_2_ amendment, 50ºC | 812,980 | 42,538 | 91 | 27 | N/A | 1/15/14 | 0 |
| *Halomonas* sp. SCGC AD-702-P13 | 2616644911 | NO_3_ and H_2_ amendment, 50ºC | 298,520 | 49,783 | 28 | 53 | 12.5 | 1/15/14 | 0 |
| *Nautilia* sp. SCGC AD-702-P16 | 2616644912 | NO_3_ and H_2_ amendment, 50ºC | 1,229,711 | 101,730 | 95 | 30 | 75.8 | 1/15/14 | 2 |
| Archaeon SCGC AD-706-D03 | 2634166880 | Control | 465,732 | 45,523 | 52 | 36 | 45.1 | 1/15/14 | 1 |
| Bacterium SCGC AD-706-E05 | 2634166881 | Control | 1,140,053 | 86,286 | 92 | 36 | 29.6 | 1/15/14 | 0 |
| Bacterium SCGC AD-706-E13 | 2634166882 | Control | 304,817 | 27,125 | 38 | 30 | 9.9 | 1/15/14 | 0 |
| *Sulfurovum sp.* SCGC AD-706-L15 | 2616644913 | Control | 366,457 | 25,877 | 45 | 34 | 28.2 | 1/15/14 | 0 |
| Bacterium SCGC AD-706-M09 | 2616644914 | Control | 562,630 | 60,582 | 43 | 33 | 10.5 | 1/15/14 | 0 |
| Bacterium SCGC AD-726-A03 | 2634166883 | In situ | 1,497,203 | 82,986 | 158 | 33 | 46.1 | 1/15/14 | 2 |
| *Arcobacer sp.* SCGC AD-726-A05 | 2616644915 | In situ | 1,013,821 | 51,899 | 136 | 28 | 49.9 | 1/15/14 | 0 |
| Bacteroidetes bacterium SCGC AD-726-B06 | 2616644916 | In situ | 1,992,123 | 82,208 | 139 | 32 | 46.7 | 1/15/14 | 1 |
| Archaeon SCGC AD-726-C03 | 2634166884 | In situ | 534,166 | 49,988 | 32 | 36 | 40.8 | 1/15/14 | 0 |
| *Thermotogae* sp. SCGC AD-726-C10 |  | In situ | 1,382,712 | 59,082 | 122 | 29 |  | 1/15/14 | 0 |
| Archaeon SCGC AD-726-C03 | 2636415966 | In situ | 679,462 | 18,372 | 151 | 23 | 45.7 | 1/15/14 | 0 |
| Gammaproteobacteria bacterium SCGC AD-726-D18 | 2616644918 | In situ | 728,624 | 52,016 | 51 | 37 | 8.6 | 1/15/14 | 0 |
| Bacterium SCGC AD-726-G11 | 2634166885 | In situ | 1,991,814 | 51,711 | 201 | 37 | 59.8 | 1/15/14 | 1 |
| Bacterium SCGC AD-726-I06 | 2634166886 | In situ | 1,079,542 | 49,679 | 125 | 28 | 62.6 | 1/15/14 | 0 |
| Archaeon SCGC AD-726-I09 | 2634166887 | In situ | 1,249,464 | 54,840 | 155 | 27 | 50.5 | 1/15/14 | 0 |
| *Sulfurovum sp.* SCGC AD-726-I20 | 2616644919 | In situ | 831,138 | 107,823 | 83 | 33 | 46.5 | 1/15/14 | 3 |
| *Sulfurovum sp.* SCGC AD-726-J17 | 2616644920 | In situ | 1,111,394 | 67,561 | 107 | 34 | 57.9 | 1/15/14 | 2 |
| Gammaproteobacteria bacterium SCGC AD-726-K05 | 2616644924 | In situ | 430,782 | 29,280 | 43 | 41 | 32.8 | 1/15/14 | 0 |
| *Sulfurovum sp.* SCGC AD-726-K20 | 2616644921 | In situ | 1,064,619 | 61,415 | 95 | 34 | 54.2 | 1/15/14 | 3 |
| Gracilibacteria bacterium SCGC AD-726-L19 | 2634166888 | In situ | 1,011,306 | 51,468 | 149 | 26 | 66.3 | 1/15/14 | 0 |
| Archaeon SCGC AD-726-L23 | 2634166889 | In situ | 655,415 | 60,449 | 58 | 31 | 50.5 | 1/15/14 | 0 |
| Parcubacteria bacterium SCGC AD-726-N03 | 2634166890 | In situ | 522,925 | 38,070 | 63 | 34 | 42.2 | 1/15/14 | 0 |
| *Sulfurovum sp.* SCGC AD-726-N15 | 2616644922 | In situ | 1,137,932 | 59,777 | 104 | 31 | 58.9 | 1/15/14 | 3 |
| *Sulfurovum sp.* SCGC AD-726-O21 | 2616644923 | In situ | 1,051,883 | 46,268 | 102 | 27 | 46.6 | 1/15/14 | 2 |
